# The complete chloroplast genome sequence of *Lirianthe hodgsonii*, a tree species of Magnoliaceae as least concern

**DOI:** 10.1080/23802359.2020.1798296

**Published:** 2020-07-29

**Authors:** Shao-Yu Chen, Tao Wu, Hui-Fen Ma, Yu-Pin Fu, Yun-Feng Zhu, Jia-Bo Hao, Hong Jiang, Yong-Kang Sima

**Affiliations:** Yunnan Academy of Forestry & Grassland Science, Kunming, People's Republic of China

**Keywords:** Magnoliaceae, *Lirianthe hodgsonii* (J. D. Hooker & Thomson) Sima & S. G. Lu, complete chloroplast genome, phylogenetic analysis

## Abstract

*Lirianthe hodgsonii* is a tree species of Magnoliaceae as least concern. In the present paper, the complete chloroplast genome (cpDNA) and basic annotated information of *L. hodgsonii* were reported and its phylogenetic relationship with other species in Magnoliaceae was analyzed. The size of its complete cpDNA is 159,693 bp, with a typical quadripartite structure comprising a pair of inverted repeat (IR) regions of 26,546 bp, a large single-copy (LSC) region of 87,848 bp and a small single-copy (SSC) region of 18,753 bp. The genome contains 131 unique genes, including 86 protein-coding genes, 37 tRNA genes, and 8 rRNA genes. The phylogenetic analysis showed that *L. hodgsonii* is affinal to *Lirianthe bidoupensis* and they form a monophyletic group with other seven *Lirianthe* species. This *Lirianthe* clade is sister to the *Dugandiodendron* and *Talauma* clade with high support. All genera mentioned in this analysis are monophyletic under the system of Magnoliaceae by Sima and Lu.

*Lirianthe hodgsonii* (J. D. Hooker & Thomson) Sima & S. G. Lu was initially described as a species of the genus *Talauma* Jussieu within the family Magnoliaceae for its circumscissile mature carpels by Hooker and Thomson (1855). They were followed by some scholars (Law and Wu 1996; Xia *et al*. 2008) although it was ever transferred to the genus *Magnolia* Linnaeus by Keng (1978). Based on further observations, Sima and Lu (2009) renamed it *Lirianthe hodgsonii* (J. D. Hooker & Thomson) Sima & S. G. Lu. The species is native to SW China (S Xizang, SE and SW Yunnan), Bangladesh, Bhutan, NE India, N Myanmar, Nepal, and Thailand (Law and Wu 1996; Xia *et al*. 2008; Kundu 2009; Nooteboom and Chalermglin 2009; Sima and Lu 2009). It is a subdominant tree species in tropical moist forest (Barua *et al*. 2018) and used medicinally (Kijjoa 2002; Nascimento *et al*. 2004). In 2007, it was evaluated as least concern in the book, *The Red List of Magnoliaceae* (Cicuzza *et al*. 2007). However, there has been no report on chloroplast genome information of *Lirianthe hodgsonii* (J. D. Hooker & Thomson) Sima & S. G. Lu until now.

In the study, the complete sequence of chloroplast genome of *Lirianthe hodgsonii* (J. D. Hooker & Thomson) Sima & S. G. Lu was reported. The GenBank accession number is MT560391. The leaf sample of a tree of *Lirianthe hodgsonii* (J. D. Hooker & Thomson) Sima & S. G. Lu was collected from Motuo County, Xizang Province of China. The sheets of vouchered specimen, H. Jiang 7337, are stored at the herbarium, YAF. Genomic DNA was extracted from dry leaves of the specimen by using DNA Plantzol Reagent (Invitrogen, Carlsbad, CA). Total genome DNA of this species was sequenced by Illumina HiSeq Sequencing System (Illumina, San Diego, CA) and shotgun library was constructed. About 2.0 Gb pair-end (150 bp) raw sequence data were obtained and the low-quality sequences were filtered using CLC Genomics Workbench v8.0 (CLC Bio, Aarhus, Denmark) to get high-quality clean reads. NOVOPlasty software (Dierckxsens *et al*. 2017) was used to align and assemble cp genome with *Pachylarnax sinica* (Y. W. Law) N. H. Xia & C. Y. Wu (JX280400) served as the reference. The complete chloroplast genome of *Lirianthe hodgsonii* (J. D. Hooker & Thomson) Sima & S. G. Lu was automatically annotated using CpGAVAS (Liu *et al*. 2012) and then adjusted and confirmed with Geneious 9.1 (Kearse *et al*. 2012). Then, the annotated genomic sequence was submitted to GenBank.

The size of complete chloroplast genome of *Lirianthe hodgsonii* (J. D. Hooker & Thomson) Sima & S. G. Lu is 159,693 bp, with a typical quadripartite structure including a large single-copy (LSC) region of 87,848 bp and a small single-copy (SSC) region of 18,753 bp separated by a pair of inverted repeat (IR) regions of 26,546 bp each. The chloroplast genome contains 131 unique genes, including 86 protein-coding genes, 37 tRNA genes, and 8 rRNA genes.

In order to determine the phylogenetic position of *Lirianthe hodgsonii* (J. D. Hooker & Thomson) Sima & S. G. Lu, 47 complete chloroplast genome sequences of the subfamily Magnolioideae from NCBI were aligned using MAFFT v. 7 (Sima and Lu 2012; Katoh and Standley 2013). Based on the system of Magnoliaceae by Sima and Lu (2012), two species of the subfamily Liriodendroideae, *Liriodendron chinense* (Hemsley) Sargent (KU170538) and *Liriodendron tulipifera* Linnaeus (DQ899947) were served as the outgroup. The maximum-likelihood (ML) tree was reconstructed with RAxML (implemented in Geneious ver.10.1 http://www.geneious.com, Kearse *et al*. 2012) and bootstrap values were calculated from 1000 replicates (Figure 1). The result of phylogenetic analysis revealed that *Lirianthe hodgsonii* (J. D. Hooker & Thomson) Sima & S. G. Lu is affinal to *Lirianthe bidoupensis* (Q. N. Vu) Sima & Hong Yu (MN990624) and formed a monophyletic group with the latter and other seven species of the genus *Lirianthe* Spach. This clade of the genus *Lirianthe* Spach is sister to the clade of the genus *Dugandiodendron* Lozano-Contreras and the genus *Talauma* Jussieu with high support. All genera mentioned in this analysis are monophyletic under the system of Magnoliaceae by Sima and Lu (2012). The determination of the complete plastid genome sequences provided new molecular data to illuminate the genus *Lirianthe* Spach in Magnoliaceae evolution.

**Figure 1.**
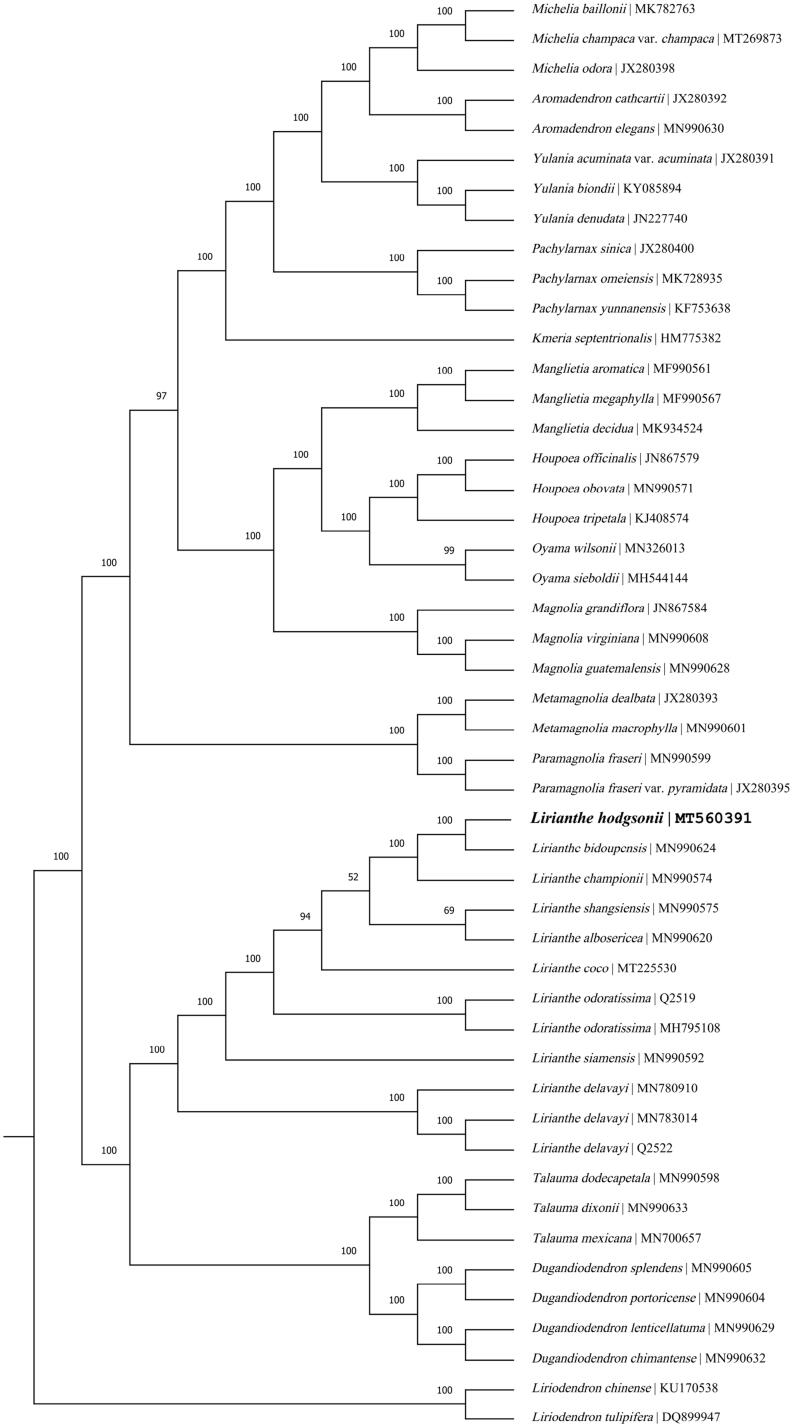
The maximum-likelihood tree inferred from the complete chloroplast genome sequences in the family Magnoliaceae. Bootstrap values (1000 replicates) are shown at the nodes.

## Data Availability

The data that support the result of this study are openly available in NCBI GenBank database (https://www.ncbi.nlm.nih.gov) and the accession number is MT560391, which permits unrestricted use, distribution, and reproduction in any medium, provided the original work is properly cited.
